# Challenges in recruitment and implementation of intervention studies including migrants

**DOI:** 10.1017/S1463423625100662

**Published:** 2025-12-29

**Authors:** Katarina Hjelm, Åsa Ernersson

**Affiliations:** 1 Department of Public Health and Caring Sciences, Uppsala University, Uppsala, Sweden; 2 Department of Health, Medicine and Caring Sciences, Division of Nursing Science and Reproductive Health, Linköping University, Linköping, Sweden

**Keywords:** barriers, intervention study, migrants, primary healthcare, recruitment, reflective notes

## Abstract

**Background:**

The health of migrants with type 2 diabetes has become a public health concern. Minority populations, including migrants, are often considered ‘hard-to-reach groups’ in clinical research, as researchers face challenges in engaging, accessing and retaining participants. Previous reviews have focused on either recruitment or retention, highlighting the need to gather experiences to obtain a more comprehensive picture for improving participation in research.

**Aim:**

To share lessons learned about the challenges of recruiting and implementing an intervention study including migrants with type 2 diabetes.

**Methods:**

This was a descriptive study, where researchers recorded experiences in reflective diaries and held discussions with the multi-professional teams involved. Data were analysed using Pawson’s conceptual framework, evaluating four dimensions of context: individual, interpersonal, institutional and infrastructural.

**Findings:**

The individual context concerns the time-consuming recruitment process since about half of the prospective participants did not want to participate, often due to illness, lack of time, the need to work, or having travelled abroad. In the interpersonal context, the main challenge was involving several professional groups; the greater the involvement, the less flexibility there was to meet expectations. The priorities in the institutional context were to provide care, with efficiency and productivity taking precedence over research. The infrastructural context was crucial due to a lack of staff available to support recruitment, the healthcare system’s burden caused by the pandemic, and the impact of laws and regulations in healthcare.

**Conclusions:**

Recruiting and implementing clinical research studies among migrant populations is complex. Factors across all contextual levels play a role, but the main challenges are within the institutional and infrastructural contexts. Changes in infrastructure influence institutional priorities, particularly with an already strained staff situation in primary healthcare. While political and social changes are difficult to alter, fostering positive attitudes towards research at the individual and interpersonal levels is important.

## Introduction

The health of migrants with type 2 diabetes has become a public health concern, particularly as this condition is increasing rapidly among migrants living in developed countries and worldwide (International Diabetes Federation (IDF), 2021). Type 2 diabetes is now considered a pandemic. Minority populations, including migrants, are often considered ‘hard-to-reach groups’ in clinical research (Metayer *et al.,* 2018), as researchers face multiple barriers to recruitment (Metayer *et al.,* 2018) and the implementation of prevention interventions (Bonevski *et al.,* 2014). Thus, gathering experiences from clinical research is vital for improving participation, especially given that migration is a growing global phenomenon, with numbers expected to rise further in the future (International Organization of Migration, 2022).

Active participation in self-care, based on knowledge about diabetes, is a cornerstone of effective health management and glycaemic control (IDF, 2021; Socialstyrelsen, 2018; ADA, 2023). Although there is ongoing debate about what kind of teaching method gives the best result, few studies have evaluated approaches specifically for migrants (Creamer *et al*., 2016; Socialstyrelsen, 2012; 2018). To address this gap, a culturally appropriate, group-based diabetes education model has been developed (Hadziabdic *et al.,* 2020). This model is tailored to both individual and cultural aspects, aiming to improve knowledge of type 2 diabetes among migrants and thus increase self-care behaviours and improve health. The present study aims to share lessons learned from the challenges of recruiting participants and implementing an intervention using this model among migrants with diabetes.

### Background

Previously, minority populations, such as migrants, have been considered ‘hard-to-reach groups’ in prevention interventions due to cultural and logistical barriers to recruitment (Metayer *et al*., 2018). Strategic, trust-based partnerships, as well as an understanding of culture and social networks, were identified as key factors in a randomized controlled obesity prevention intervention targeting new immigrant mothers and children from Latin America and Haiti within a community-based participatory research framework. Furthermore, two reviews focused on ‘hard-to-reach groups’: one examining socially disadvantaged groups, which included a limited number of migrants (Bonevski *et al.,* 2014), and another investigating approaches to broaden participation in research studies among these groups (Shaghaghi *et al.,* 2011). The latter highlighted ‘hidden populations’, defined by their physical and geographical location (e.g. forests, mountains, deserts) or their social and economic situations, including migrants. Study compliance was reported to depend on group characteristics, recruitment techniques and the subject matter, with knowledge of the target population linked to greater success. Barriers and recruitment strategies for migrants with precarious legal status have been investigated in a mixed-methods study in Canada (Fête *et al.,* 2019). The interviewers’ experience and understanding of participants’ situations, as well as the development of a community resource guide tailored to their needs, were crucial to the study’s success.

Another review investigated the complex issue of recruiting culturally and linguistically diverse (CALD) older people for medical research and examined strategies to address these issues (Hughson *et al.*, 2016). The literature indicated that predominant barriers were communication difficulties, including literacy and health literacy; English language competence; and cultural factors within the research setting, such as mistrust of consent processes. Practical and logistical barriers, including mobility limitations, were also highlighted.

Researchers continue to face challenges in engaging, accessing and retaining participants from socially disadvantaged groups. Previous reviews have mainly focused on one aspect of the research process: either recruitment or retention, thus failing to provide a complete picture (Bonevski *et al*., 2014). This highlights the need to gather experiences from clinical research to develop a more comprehensive understanding of strategies for improving participation. Notably, the literature review did not reveal any studies focusing on the challenges of recruiting and implementing intervention studies involving migrants with diabetes, particularly within the context of primary healthcare.

## Method

### Design

This descriptive study is based on experiences and reflections from the recruitment and implementation of the intervention study, recorded by the project leader/principal investigator (PI, first author) and the assisting postdoctoral researcher (second author), along with discussions with the multi-professional team members involved. Throughout the research process, reflective notes were taken on challenges and possibilities. These notes formed a steadily growing reflective diary, documenting project progress and lessons learned (Patton, 2015).

**Figure 1.**
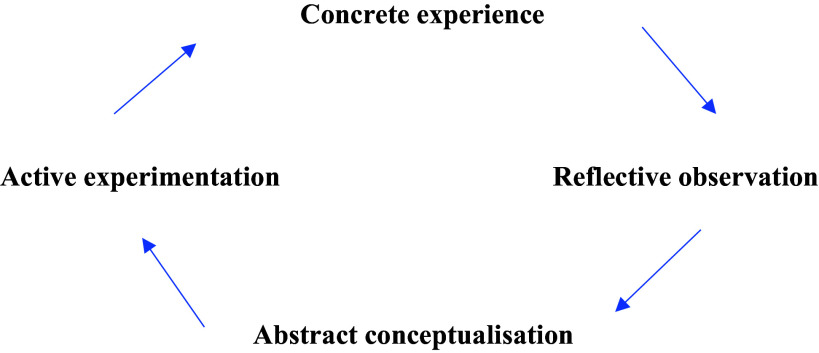
Processing free after Kolb’s experiential learning cycle (Kolb, 1984).

Kolb’s Experiential Learning Cycle (Kolb, 1984) was used as the reflective framework for this study (see Figure 1). The model centres on developing understanding through actual experiences and comprises four key stages: Concrete experience, reflective observation, abstract conceptualization, and active experimentation. The model describes that we start with an experience, either a repetition of something that has happened before or, as in this case, something completely new to us. In the next stage, we reflect on the experience and note anything that is unfamiliar. This reflection leads to the development of new ideas, e.g. when something unexpected has happened, we try to find out why. Finally, these new ideas are applied in different situations. Learning is, thus, a direct result of our experiences and reflections.

### Analysis framework

We utilized Pawson’s (2013) theoretical framework as the basis for analysing and presenting the data, focusing on the science of evaluation and the dimensions of context. Four contexts are described: (1) individual, (2) interpersonal, (3) institutional, and (4) infrastructural. The interpersonal context refers to relationships among team members, both in the research team and the multidisciplinary diabetes team within the studied clinic, as well as the migrant participants. The institutional context relates to the research setting, i.e. the healthcare centre where the study was conducted. Lastly, the infrastructural context refers to the social and cultural environment of the organization, including laws and regulations relating to healthcare, as well as changes in societal health patterns.

### The intervention study that was a subject of reflection

#### Setting

The intervention study, which provided the focus for collecting experiences and reflections in this descriptive study, was conducted in primary healthcare centres within a municipality in central Sweden, with approximately 242,100 inhabitants. Of these, 55,700 individuals, that is, 23%, were born abroad (Statistics Sweden, 2022). In 2015, Sweden experienced its highest influx of refugees ever (Statistics Sweden, 2016). The newcomers mainly originated from Afghanistan, North Africa, and Syria. They were added to the migrant population already living in Sweden, composed largely of labour migrants who arrived during the post-war era and the 1970s, from Finland and ex-Yugoslavia. From mid-1985 onwards, immigration patterns changed to include mainly refugees, first from Latin America, Asia, and Africa, and later, during the 1990s, from conflicts in ex-Yugoslavia and the Middle East. Thus, the migrant population is a mix of nationalities (>140). Foreign-born individuals account for about 20% of Sweden’s population of 10 million (Statistics Sweden, 2022), forming a heterogeneous and multilingual group with vast differences in language and cultural backgrounds.

The healthcare centres selected for this study are situated in the most densely populated immigrant areas (60% [second generation included] of the municipality. In Sweden, primary healthcare is the basis of the healthcare system and is delivered through healthcare centres with outpatient clinics staffed by general practitioners (GPs), nurses, and assistant nurses. Each centre serves a defined population based on locality and access criteria. Primary healthcare is responsible for public health and for treating conditions that do not require hospital or specialist care. Patients needing specialized care are referred to hospitals, with university hospitals offering the most highly specialized care (SOU 2020:19). In this case, the selected healthcare centres were located in a region with access to one of the country’s six academic university hospitals.

#### Description of the intervention

The diabetes education model tested in the intervention was developed to increase knowledge about diabetes and influence self-care among migrants diagnosed with type 2 diabetes. Specifically, it is a culturally appropriate model that builds on individual beliefs about health and illness, based on knowledge, and is delivered through focus group discussions comprising five sessions. Sessions are led by a diabetes specialist nurse (DSN) in collaboration with a multi-professional team and are completed within three months. The different members of the diabetes team (nurse, physician, and dietician) participate according to the themes planned for each session. Each focus group includes 4–5 participants and lasts approximately 90 minutes, in the presence of a gender-matched interpreter. A thematic interview guide with broad, open-ended questions and descriptions of critical situations/health problems is used. Participants are encouraged to discuss their individual beliefs and knowledge, while healthcare staff provide answers, additional information, and ensure that basic principles of diabetes care are addressed when necessary. The model is tailored to both individual and cultural aspects, aiming to improve participants’ knowledge of type 2 diabetes, thus increasing self-care behaviours and improving health (see Hadziabdic *et al*., 2020).

The effects of the intervention are evaluated through semi-structured interviews and measurements of HbA1c at baseline (before the start) and immediately after completion, as well as at three months, one year, and three years after the intervention. Focus group interviews are conducted directly after completion to evaluate participants’ experiences of participation.

#### Description of participant recruitment for the intervention

Study participants were invited to participate in the intervention study by staff at the healthcare centres. After obtaining written consent from the heads of the centres and approval from the National Ethical Review Board, the research team attended workplace meetings with staff, including the diabetes care team, to provide both oral and written information about the study and the recruitment process. A DSN at each diabetes clinic was appointed by the management to oversee recruitment and serve as the main contact person for staff, patients, and researchers. The DSN received additional individual briefings from the researchers. Further information about the project was provided and discussed with the PI during regular lunch seminars.

Eligible patients were mainly invited by the DSN, using translated written information. A reply slip was filled in and forwarded to the researcher, who then contacted the participant to arrange the first meeting. This meeting was held in a secluded room at the healthcare centre. After obtaining written informed consent, baseline data were collected through an interview (about one hour), followed by an HbA1C test. Thereafter, a date was set for the first group education session. During this session, a time plan for the following sessions was set, based on a proposed schedule determined by the availability of the staff in the multi-professional diabetes team. Efforts were made to meet on the same day and at the same time every second week, usually at the end of the day before the healthcare centre closed (15:00–16:30). All expenses related to staffing needs during the recruitment, implementation of group education, and blood tests were covered by the project budget.

#### Description of the intervention implementation

The intervention study started in autumn 2019 and ended in October 2023. However, the study period was significantly affected by the Covid-19 pandemic, from March 2020 to May 2022. During this time, the study was paused on three occasions (at the start, middle, and end phases) due to healthcare restrictions on in-person visits, which did not allow for group education sessions. As a result, no new groups could be initiated. Once restrictions were lifted, the group sessions that later were started were completed as planned without further interruption.

Individuals with diabetes, considered a high-risk group, were subject to stricter restrictions to protect them against infection. Throughout these years, communication with healthcare staff continued through telephone calls, e-mail and digital meetings (Zoom, Teams, Skype). In the post-Covid phase, during autumn 2022, the intervention was restarted for the fourth time. Staff in the healthcare centres expressed being highly motivated to resume the study, but at the same time, they described feeling heavily burdened by the backlog of deferred treatments caused by the pandemic. This was attributed to the perceived overzealous focus among healthcare staff on productivity, which further delayed and halted the study once more.

## Results

### Individual context

Several challenges emerged during the recruitment process, which proved both time-consuming and difficult. Many prospective participants spent a large part of the year abroad, which made it difficult to reach them by phone or book appointments. Although written information about the study was distributed, including a specified time for telephone contact, the majority did not answer the phone calls. When contact was established through telephone, more information about the study was provided; however, most prospective participants refused to take part. Often, this was due to health concerns or work commitments, which meant they could not take time off.

Further challenges were experienced in terms of attrition rate. Of those who initially agreed to participate and were scheduled for the first appointment, namely the first interview, approximately 50% did not attend. When contacted again by phone to book a new appointment, the majority had changed their minds and did not want to participate any longer. Some cited reasons such as their own or a relative’s illness, or having been scheduled for an operation, while others provided no explanation. Some participants completed the baseline interview and started attending the educational sessions but did not participate consistently. Late arrivals were also common, often attributed to lack of time, the need to work, being tired or having travelled abroad. Many prospective participants spent extended periods outside of Sweden, which made it difficult to reach them by phone to book and/or change appointments. For the same reason, conducting long-term follow-ups within the prescribed time intervals was difficult to perform.

### Interpersonal context

Coordination and communication with designated contacts at the healthcare centres generally worked well when arranging individual meetings. However, booking group sessions proved more challenging, as it required coordination among several professional groups, which reduced overall flexibility. Thus, the greater the number of professionals involved, the more difficult it became to accommodate participants’ expectations and availability.

Recruitment was further challenged due to the multiple contacts through another person, often a relative or friend, who answered the phone and in turn had to relay the information. This additional step meant that contact could not always be established directly with the prospective participants. An interpreter was available for all planned telephone contacts, and in the cases where direct contact was established, communication worked effectively.

Expressions of dissatisfaction with the healthcare provided, particularly diabetes care, could be noted during some sessions. This dissatisfaction, which was expressed both verbally and through body language, fostered a sense of frustration among both participants and team members during the sessions. Additionally, some team members faced difficulties in transitioning from a professional role, focused on delivering information, to adopting a more person-centred approach that meant listening to individual beliefs, addressing perceived obstacles, and understanding participants’ motivational needs.

### Institutional context

It was a challenge to include new healthcare centres to carry out the research project. During the first contact with managers and diabetes nurses, there was often a positive attitude towards participation, with several perceived advantages in carrying out an educational programme developed for migrants with type 2 diabetes. However, there were often long delays before receiving a decision regarding whether the study could commence, and often, the responses were negative, requiring the process to start over. There were various reasons for healthcare centres not participating in the research project. These ranged from prior commitments to other research projects, or sick leaves in the staff group, pregnancy, or planning for parental leave, and overall high workload. Occasionally, even if the staff were interested, the operation’s manager decided that they must primarily prioritize care, efficiency and productivity before research activities. Consequently, recruitment to the project stopped in certain healthcare centres due to time constraints and the perceived pressure among staff to prioritize clinical duties over research involvement.

### Infrastructural context

Currently, we are facing challenges due to a shortage of staff available to assist with recruitment, a backlog of care resulting from the pandemic, and laws and regulations governing the activities researchers are permitted to undertake within healthcare, particularly in relation to recruiting participants.

According to Swedish law, only healthcare centre staff may access patients’ contact information in the medical records. This made the recruitment process for prospective participants more difficult and caused delays, as the DSN was required to forward contact information to the research nurse, who could then continue the recruitment process.

Another challenge was that the recorded contact information for prospective participants in the digitalized medical record often belonged to another person or was outdated. Relatives or friends often handled all contacts with healthcare due to language difficulties, making direct contact with prospective participants difficult. Although an authorized interpreter was available at all scheduled phone calls, making spontaneous calls was challenging, as interpreters needed to be booked in advance. These factors further delayed the recruitment process.

The implementation of the intervention and group education sessions was temporarily halted from March 2020 to March 2022 due to Covid-19 restrictions. The recruitment process and planning were significantly affected by uncertainty over whether the intervention could proceed or would be paused once more. Moreover, prospective participants were hesitant to enrol due to the ongoing restrictions, which persisted to some extent. This hesitancy may also be attributed to signs of burnout among staff, following the high demands during and after the pandemic. Thus, currently, staff requested a pause in running the intervention groups and has desired/proposed to start again later this spring or in the autumn. However, efforts to extend the study to other healthcare centres have been met with resistance, as staff prioritized their own work and well-being over participation.

## Discussion

This study is unique as it shares the lessons learned from challenges in recruiting and implementing an intervention study involving migrants with diabetes. By capturing experiences throughout the whole research process, rather than focusing on parts of it as in previous studies, it provides a more comprehensive understanding of strategies to improve participation in research. Consequently, direct comparisons with earlier studies are only partially applicable.

The main finding is that the infrastructural context is of great importance for the implementation of research within healthcare centres, as the conditions for conducting research are largely governed by this context, as well as by the institutional context. These two contexts together create the conditions for research to take place. While the individual and interpersonal contexts influence the success of an intervention like the one planned in this study, the infrastructural and institutional contexts form the essential basis that determines whether implementation is feasible at all (Pawson, 2013).

As previously noted (Bonevski *et al*., 2014; Metayer *et al*., 2018; Fête *et al.,* 2019), migrants are a hard-to-reach group. However, in contrast to other studies, it was surprising to find challenges in identifying potential participants and obtaining accurate, up-to-date contact details (name, address, telephone number), despite the use of digitalized medical records. Many individuals often move within and outside the country, leading to changes in address, telephone numbers, or mobile devices. Others provide contact information for third persons (often Swedish-speaking cohabitants), which affects the accuracy of the records.

Thus, measures are needed at the institutional level (Pawson, 2013) to adapt modern digitalized systems to everyday practice. Digitalization is crucial for developing sustainable healthcare systems (SOU, 2020). Furthermore, the influence of the infrastructural context (Pawson, 2013) regarding existing laws on secrecy/non-disclosure and confidentiality (SFS 2009:400; SFS 2010:659) has not been previously discussed. These laws provide a framework to protect individuals; thus, only healthcare staff are allowed to contact patients for research purposes. As most researchers are employed by universities rather than healthcare organizations, this can be problematic. Streamlined employment processes (based on project budgets) should be prioritized in planning studies. Moreover, healthcare leaders should receive training on how to navigate these regulatory frameworks.

Other problems mentioned in both the recruitment and implementation phases included attrition and retention issues. The studied population, patients in primary healthcare, seemed to differ from those in previous research (Hjelm *et al.,* 2003; 2005), as their information-seeking behaviour was not disclosed. In addition, interpreters spontaneously reflected on some respondents expressing feelings of being too old and having a fatalistic view of their health and life, which appeared to reduce motivation and engagement. These differences may relate to beliefs about health and illness (Hjelm *et al.,* 2003; 2005), health literacy levels (Hughson *et al.,* 2016), lower risk awareness due to limited knowledge (Pettersson *et al.,* 2019), as well as factors such as educational level, migration background, present health status and prior experience with specialized diabetes care (Hjelm *et al.,* 2003; 2005).

The content, extent and scope of the interviews (about 45 minutes) were additional factors (Patton, 2015) that may have contributed, as participants could have been confronted with gaps in their own knowledge, leading some to withdraw from the study. Other priorities in daily life (Bonevski *et al.,* 2014), such as time constraints connected with family and job responsibilities (Hughson *et al.,* 2016) and travel abroad, further influenced participation. In autumn 2023, the surrounding political crises in the world (e.g. conflict in Gaza) and negative discourse about Sweden in countries in the Middle East (e.g. Iraq) seemed to affect the mood of the respondents and attitudes towards the researcher/research. Thus, a combination of factors related to the individual, interpersonal, and infrastructural contexts influenced participation (Pawson, 2013).

During the implementation phase, numerous factors influenced the process at the individual, interpersonal, institutional and infrastructural levels (Pawson, 2013). Not only was recruitment challenging, but implementation was also considered time-consuming, mainly due to the Covid-19 pandemic and its aftermath. Heavy workloads, staff shortages, exhaustion (Mughal *et al.,* 2021; Russo *et al.,* 2023), and frustration resulting from the study being paused three times had a significant impact, leading to staff burnout. The accumulation of care backlogs (Mughal *et al.,* 2021; Russo *et al.,* 2023) also forced leaders/managers to halt the study, despite respondents having undergone baseline measurements. Productivity aspects were prioritized, resulting in ethical conflicts for both staff and researchers, leaving unsolved questions about how to tackle these challenges.

The generalizability of the results may also have been affected by the repeated disruptions due to the pandemic. However, in this case, the disruptions meant that no new groups could be started during these periods, but those sessions that had already started were completed without further interruption.

Interpersonal issues further influenced continuation (Pawson, 2013), as some team members found it difficult to adapt their professional roles to the new person-centred pedagogical approach. Shifting from traditional information delivery to actively listening to and addressing participants’ beliefs, obstacles and motivational needs often conflicted with established biomedical educational practices (Stenov *et al.,* 2019). Tailoring content and processes to meet the needs of group participants was perceived as difficult, and there was reluctance to use customized educational materials. Thus, the combination of these factors led to team members influencing each other, often asking themselves, ‘what’s in it for me?’ They were unable to understand the long-term perspective required for conducting research (Bonevski *et al.,* 2014).

### Strengths and limitations

Collecting experiences and reflections over a time span may introduce recall bias (Patton, 2015). However, different forms of triangulation were applied to increase the trustworthiness and validity of the findings (Patton, 2015). These forms included investigator triangulation, with two researchers sharing/comparing experiences; theoretical triangulation, using Pawson’s (2013) framework for data analysis; and methodological triangulation, drawing on different sources for data collection such as reflective notes, discussions with key persons, etc. However, it is important to note that perspectives from non-responders and, in part, non-attenders are lacking. Although these insights would have been valuable, they could not be collected due to the legal and ethical restrictions regarding confidentiality and data protection. This limitation will be partly addressed in a forthcoming study evaluating participation from the perspective of those who attended the intervention.

### Implications

The lessons learned from the challenges faced in recruiting and implementing this study highlight several implications. First, sharing information widely through different channels is essential to increase awareness and motivate participation. This includes addressing mistrust of consent procedures and overcoming communication barriers in terms of literacy and health literacy (Hughson *et al.,* 2016). The use of media resources, e.g. radio, television, newspapers, as well as collaboration with community organizations, such as associations for immigrants, can help build trust and strengthen relationships (Bonevski *et al.,* 2014). These strategies also allow the study and its health implications to be framed in a positive way, further contributing to increased participation and retention rates. They also provide opportunities to give collaborating groups feedback on the study’s progress. To facilitate recruitment within the legal framework governing non-disclosure and confidentiality in healthcare, opportunities should be created for researchers to be employed directly within the healthcare institution where the study is based. It is therefore important for leaders to understand these rules in relation to the need for research and development in primary healthcare, and this understanding should be part of their training, as they are key persons in the process. Establishing research centres or fostering research collaboration dedicated to developing high-quality, evidence-based healthcare is a way of promoting a strong research culture that is needed in today’s primary healthcare (Bonevski *et al.,* 2014). In a situation with a highly constrained healthcare budget, resources should be allocated towards developing the most effective and high-quality care at the lowest possible cost, benefiting patients, staff and society (SOU, 2020:19; Russo *et al*., 2023).

Training and capacity-building for healthcare leaders and staff are important tasks (Bonevski *et al*., 2014). Furthermore, in all kinds of training for healthcare staff, both at the graduate and postgraduate level, as well as in continuous medical education (CME) throughout their working life, the importance of participating in research and development work as part of daily work should be emphasized. This helps foster a culture of openness, curiosity and responsibility towards research/development. Joint training initiatives involving students and clinically active staff are also important tasks in promoting this culture.

## Conclusions

The experiences of recruiting and implementing an intervention involving migrants with diabetes in primary healthcare show that this is a complex process. Various factors in the different contexts, such as the Covid-19 pandemic, the workload in primary care and political/social changes in the world, interact with infrastructural and institutional conditions as well as individual and interpersonal factors, influencing attitudes towards health, illness, and everyday work, and influencing motivation among healthcare staff. We hope this study will stimulate further debate on development and research in primary healthcare.
